# Evaluation of Pulmonary Functions in Patients With Type 2 Diabetes Mellitus: A Cross-Sectional Study

**DOI:** 10.7759/cureus.35628

**Published:** 2023-03-01

**Authors:** Swati Mittal, Manisha Jindal, Saurabh Srivastava, Smriti Sinha

**Affiliations:** 1 Physiology, All India Institute Of Medical Science, Deoghar, IND; 2 Physiology, School of Medical Sciences & Research, Sharda University, Greater Noida, IND; 3 Internal Medicine, Government Institute of Medical Science, Greater Noida, IND; 4 Physiology, Mamta Academy of Medical Sciences, Hyderabad, IND

**Keywords:** restrictive pattern, pulmonary function test, forced expiratory volume in first second, forced vital capacity, diabetes mellitus

## Abstract

Introduction

Diabetes mellitus (DM) has been broadly recognized as the syndrome of hyperglycemia leading to various macro- and microvascular complications. The different physiological systems that have been identified as a target of these injurious effects of hyperglycemia are the excretory system, ocular system, central nervous system, and cardiovascular system. To date, not much focus has been given to the respiratory system as a possible target for the deleterious effect of hyperglycemia.

Objective

To assess the pulmonary functions in subjects with type 2 diabetes mellitus (T2DM) and compare them with age and sex-matched healthy controls.

Methods

This study was conducted on one hundred and twenty-five patients with type 2 diabetes mellitus and a comparative number of age and sex-matched non-diabetic individuals (control group) who met the inclusion and exclusion criteria. RMS Helios 401 computerized spirometer was used to assess pulmonary functions.

Results

The mean age of the control group and type 2 diabetics were 50.96±6.85 and 51.47±8.43 years, respectively. The results of the present study showed significantly lower values of FVC, FEV1, FEF25-75%, and MVV among diabetic subjects as compared to controls (<0.05).

Conclusion

We found that pulmonary function parameters in diabetic subjects were consistently lower than in healthy controls. This reduction in lung function is probably a chronic complication of type 2 diabetes mellitus.

## Introduction

Diabetes mellitus is becoming an increasingly prevalent metabolic disorder with ramified effects on several body systems mainly co-related with effects on micro and macrovascular circulation. The complications have been associated with increased morbidity and mortality due to their effects on different organs. Most of the earlier studies have focused on renal, ocular, and cardiac effects; however, the focus has now shifted to the involvement of other systems. The International Diabetes Federation estimated a global prevalence of diabetes at 425 million in 2017 out of which India had 73 million adults suffering from diabetes. This was estimated to rise to 134 million adults by 2045 [1].

Since the global burden of diabetes is expected to rise further in the coming years, it is imperative that more studies are conducted to ascertain the effect of this multi-systemic disease on pulmonary functions as well. Lungs and airways have a rich blood supply that contributes to approximately ten percent of the entire circulatory system of the human body. It is also known that chronic hyperglycemia causes non-enzymatic glycosylation of proteins such as collagen, elastin, etc., which leads to the thickening of the basement membrane and microangiopathy. As diabetes mellitus has been proven to have detrimental effects on the microvasculature, it is quite probable that pulmonary functions may be affected in diabetes mellitus [2,3].

Although several studies have been conducted in the recent past to assess the deleterious effects of T2DM on lung functions, nothing has been strongly established so far and related literature in India is limited. Hence, this study was conducted to determine the effect of type 2 DM on pulmonary function tests.

This article was previously posted to the research square preprint server on February 25, 2022.

## Materials and methods

A cross-sectional study was conducted in the departments of Physiology and Medicine, School of Medical Sciences and Research, Sharda Hospital, Greater Noida, Uttar Pradesh, India among subjects of type 2 diabetes mellitus. After obtaining ethical clearance for the study from the Institutional Ethical Committee of the School of Medical Sciences and Research (Ref. No. SU/SMS&R/76-A/2021/114) and ensuring that all inclusion criteria were met, one hundred and twenty-five subjects of type 2 diabetes Mellitus of either sex in the age group of 35-65 years with a duration of diabetes > 1 year were randomly selected from the out-patient department (OPD) of Sharda Hospital. Approximately, the same number of age- and sex-matched healthy individuals were selected as the control group. Informed consent was taken from each subject for the study.

Exclusion criteria

Subjects with a history of smoking (current, ex-smoker, and passive smokers), acute or chronic respiratory disease, history of occupational exposure to respiratory deterrents, neuromuscular or cardiovascular diseases, or any physical disability that may affect lung function like kyphoscoliosis and inability to perform pulmonary function tests were excluded from the study. 

Sampling procedure

To determine the sample size, the Cochran formula: n = Z2PQ/d2 was used, where, Z = 1.96, P for prevalence was taken from a previous study [4], i.e., 0.087; Q = 1 - 0.087, and d = allowable error i.e.,5% or 0.05. Thus, the calculated sample size was n = 121.

Procedure

After providing the relevant information about the study, the subjects were asked to fill out the pre-prepared questionnaire regarding relevant personal, sociodemographic, and medical history.

General and systemic examination as well as anthropometric measurements were done on both, the diabetic mellitus subjects and the control group, and Body Mass Index (BMI) was calculated by the formula of weight (kg) /height (m2).

Pulmonary function test

The pulmonary function test of the control group (Group I) and diabetes mellitus subjects (Group II) were performed by using RMS Helios 401 computerized spirometer (Recorders & Medicare Systems Pvt Ltd (RMS), India), according to American Thoracic Society/European Respiratory Society (ATS/ERS guidelines) [5,6]. Control and diabetic subjects performed the maneuver three times at the interval of 15 minutes, and the best of the three was taken for analysis. Parameters assessed were - forced vital capacity (FVC) in liters, forced expiratory volume in 1 second (FEV1), FEV1/FVC in percentage (%), forced expiratory flow during 25-75% of FVC (FEF25-75), slow vital capacity (SVC), and the maximum ventilatory volume (MVV). For all these parameters, the percentage of the predicted values for the respective age, height, and weight were taken into consideration.

Statistical analysis

Statistical analysis was carried out using the statistical software SPSS (Statistical Package for Social Sciences). Mean and standard deviation were computed for all continuous variables and comparison was done using Student's t-test. Frequencies were generated for categorical variables and compared using the chi-square test, and a p-value of <0.05 was considered as statistically significant.

## Results

A total of two hundred thirty-nine subjects were included in this study, out of which 125 were subjects with diabetes mellitus and 114 controls.

The mean age of the control group and type 2 diabetics were 50.96±6.85 and 51.47±8.43 years, respectively. Demographic characteristics (Age, Height, Weight, and BMI) of the control group (Group I) and diabetes mellitus subjects (Group II) are given in table 1.

**Table 1:** Demographic characteristics of subjects

Parameters	Group I (114)	Group II (125)	p-value
Age (years)	50.96 ± 6.85	51.47 ± 8.43	0.35
Height (cm)	163.08 ± 11.55	164.86 ± 10.39	0.18
Weight (kg)	66.73 ± 11.50	71.49 ± 11.33	0.008
BMI (kg/m^2^)	25.09 ± 3.64	26.12 ± 3.82	0.05
Gender	Male – 61 Female - 53	Male – 66 Female - 59	

The mean value of HbA1c in the diabetic group was 6.63 ± 1.05, and 4.45 ± 0.58 in the control group. In the participants with type 2 diabetes, 9.6% (12) had high serum creatinine levels (> 2mg/dl). The ALT was elevated in 32% (40) of diabetic subjects while 38.4% (48) participants showed a deranged lipid profile.

The mean Pulmonary Function Test recording among diabetic subjects and the control group has been given in Table 2. Our results show significantly lower values of FVC, FEV1, FEF25-75%, and MVV, and higher values of FEV1/FVC among diabetes mellitus subjects (<0.05). Values of slow vital capacity (SVC) were lower among the diabetes mellitus subjects as compared to the control group. However, this difference was not statistically significant.

**Table 2:** Comparison of pulmonary function test parameters between Group I and Group II FVC: Forced Vital Capacity, FEV1: Forced Expiratory Volume in 1 second , FEV1/FVC in percentage (%), FEF25-75% : Forced Expiratory Flow during 25–75% of FVC, SVC: Slow Vital Capacity,  MVV: Maximum Ventilatory Volume.

S.No.	PFT Parameters	Group I (N=114)	Group II (N=125)	p-value
1	FVC	2.47 ± 0 .65	1.95 ± 0 .95	0.0001
2	FEV_1_	1.91 ± 0 .49	1.42 ± 0.81	0.0001
3	FEV_1_/FVC	81.70 ± 3.17	98.75 ± 13.11	0.0001
4	FEF _25-75%_	2.48 ± 0 .60	1.65 ± 1.01	0.0001
5	SVC	2.49 ± 0.64	2.32 ± 0.77	0.08
6	MVV	91.01 ± 7.29	66.82 ± 18.16	0.0001

From the observed respiratory pattern (criteria are mentioned in the methodology), it was found that although the control group subjects mostly had a normal pattern of lung function, subjects in the diabetic group had a restrictive pattern of lung function in most cases. A mixed pattern was also observed in 8.9% of cases. The respiratory pattern in the two groups has been depicted in Table 3. 

**Table 3:** Respiratory pattern between Group I & Group II

Pulmonary function defects	Group I (N=114)	%	Group II (N=125)	%	Chi Square	p-Value
Restrictive	2	1.8	73	58.4	78.31	0.0001
Obstructive	7	6.1	10	8.0	0.301	0.583
Mixed pattern	4	3.5	11	8.8	3.585	0.058
Normal	101	88.5	31	24.8	92.047	0.0001

## Discussion

The results of this study show a significant decline in the values of most parameters of pulmonary function tests except FEV1/FVC among type 2 diabetes mellitus subjects (p<0.05) as compared to healthy controls. Moreover, the maximum decline was seen in FVC, FEV1, and MVV values. FEV1/FVC was significantly higher in patients with diabetes mellitus as compared to controls indicating a restrictive pattern of pulmonary functions in diabetic individuals. Although there was a decline in SVC also among diabetic subjects, the results were not statistically significant as compared to the control group. Some of the early studies have shown similar results, namely low vital capacity and restrictive pattern in type 2 DM.

Davis et al conducted a prospective study in Europid patients of type 2 DM and revealed that spirometry measures continued to decline at an annual rate of 68, 71, and 84ml/year and 17l/min for FVC, FEV1, VC, and PEF, respectively. When expressed as a percent predicted value, the means of all spirometry measures were reduced by >9.5% [7]. 

The results of this study were in agreement with the study of Lange et al, who found that there was a significant reduction in lung function in diabetic subjects as compared to control subjects and it was more prominent in subjects treated with insulin than in subjects treated with oral hypoglycemic agents and/or diet [8].

Recent studies conducted by Piyush et al [9], Shah et al [10], and Aparna [11] concluded that mean values of FVC, FEV1, and FEF25-75%, were significantly reduced in patients of type 2 DM. Additionally, Keerthi et al [12] also mentioned the reduction in MVV values in diabetic subjects. 

The finding of reduced ventilatory function and a restrictive pattern is in accordance with the studies conducted by Anandhalakshmi et al and Goya et al [13,14]. Another study done by Walter et al [15] showed that FEV1/FVC% was significantly increased by 1.5% in patients with type 2 DM. A meta-analysis by Borst et al mentioned that DM is associated with a restrictive pattern of pulmonary function [16].

On the contrary, Benbassat et al [17] and Sandler et al, [18] failed to observe a significant association between type 2 DM and abnormal pulmonary function parameters. This is probably because Benbassat et al had not compared their results with a matched control group, and Sandlet et al had studied a small sample size.

Conversely, a study done by Cazzola et al on human isolated bronchi demonstrated the obstructive pattern of pulmonary pathology in patients with diabetes mellitus [19]. Rajan et al determined that spirometry readings in the Indian diabetic population showed a variable pattern with 60% obstructive, 30% restrictive, and 23% mixed pattern [20].

The pathophysiological correlation of diabetes mellitus with deteriorated pulmonary function may be multifactorial. The lung volume comprises parenchyma which includes a large number of alveoli and non-parenchymal structures comprised of pulmonary vessels and bronchial tree [21]. Proper functioning of both these components is required for optimal lung function. Airways, blood vessels, and the interstitium of the lung parenchyma are rich in various types of collagens and elastin. The non-enzymatic glycosylation of these structural proteins leads to alteration in functions of lung connective tissue and stiffening of the chest wall which may be the potential mechanism for mechanical lung dysfunction in diabetes mellitus [22].

Thickening of the basal lamina of capillaries is seen in patients with diabetes mellitus and has also been documented by some human postmortem studies [23]. This may result in a redistribution of vascular flow with reduced perfusion in well-ventilated areas leading to a decline in lung function [18]. Patients of diabetes mellitus also had neuropathy leading to neuromuscular dysfunction which adversely affects pulmonary function [24]. Low-grade inflammation in diabetics adds to the deleterious effects of the disease on lung function.

A combination of all these factors continues to have a hazardous effect on pulmonary functions resulting in their decline and is a cause of concern that is not yet truly understood by clinicians. In the context of growing attention in the clinical use of inhaled insulin, it is the need of global health care to understand the link between insulin and lung function. Hence more detailed histopathological studies are required for this treatment option to be taken into consideration

Limitations and future perspective

Diabetes mellitus is a widely prevalent disease and hence longitudinal studies with more patients need to be conducted. Diffusion studies were not performed in this study. This study has not worked on the association between glycemic control and pulmonary function. It is intriguing to think of the lung as another end organ adversely affected by diabetes. Lung function needs to be monitored periodically to evaluate the severity of impairment. 

## Conclusions

The present study demonstrates that the lungs should also be considered as a primary target organ for diabetic complications along with other micro- and macrovascular complications. There was a significant decrease in FVC, FEV1, FEF 25-75%, and MVV, and a dominant restrictive pattern of pulmonary dysfunction was observed in subjects of type 2 diabetes mellitus as compared to non-diabetic healthy controls. The exact pathophysiology for reduced pulmonary functions in diabetes mellitus is still an interesting area of research and more prospective studies in larger populations should be performed using advanced techniques to include this as a long-term complication of diabetes mellitus.
